# Hemicelluloses negatively affect lignocellulose crystallinity for high biomass digestibility under NaOH and H_2_SO_4_ pretreatments in *Miscanthus*

**DOI:** 10.1186/1754-6834-5-58

**Published:** 2012-08-11

**Authors:** Ning Xu, Wei Zhang, Shuangfeng Ren, Fei Liu, Chunqiao Zhao, Haofeng Liao, Zhengdan Xu, Jiangfeng Huang, Qing Li, Yuanyuan Tu, Bin Yu, Yanting Wang, Jianxiong Jiang, Jingping Qin, Liangcai Peng

**Affiliations:** 1 National Key Laboratory of Crop Genetic Improvement and National Centre of Plant Gene Research, Huazhong Agricultural University, Wuhan, 430070, China; 2 Biomass and Bioenergy Research Centre, Huazhong Agricultural University, Wuhan, 430070, China; 3 College of Plant Science and Technology, Huazhong Agricultural University, Wuhan, 430070, China; 4 College of Life Science and Technology, Huazhong Agricultural University, Wuhan, 430070, China; 5 College of Science, Huazhong Agricultural University, Wuhan, 430070, China; 6 Department of Biotechnology, Hunan Agricultural University, Changsha, 410128, China; 7 Jiangsu Key Laboratory for Biomass-based Energy and Enzyme Technology, Huaiyin Normal University, Huaian, 223300, China

## Abstract

**Background:**

Lignocellulose is the most abundant biomass on earth. However, biomass recalcitrance has become a major factor affecting biofuel production. Although cellulose crystallinity significantly influences biomass saccharification, little is known about the impact of three major wall polymers on cellulose crystallization. In this study, we selected six typical pairs of *Miscanthus* samples that presented different cell wall compositions, and then compared their cellulose crystallinity and biomass digestibility after various chemical pretreatments.

**Results:**

A *Miscanthus* sample with a high hemicelluloses level was determined to have a relatively low cellulose crystallinity index (CrI) and enhanced biomass digestibility at similar rates after pretreatments of NaOH and H_2_SO_4_ with three concentrations. By contrast, a *Miscanthus* sample with a high cellulose or lignin level showed increased CrI and low biomass saccharification, particularly after H_2_SO_4_ pretreatment. Correlation analysis revealed that the cellulose CrI negatively affected biomass digestion. Increased hemicelluloses level by 25% or decreased cellulose and lignin contents by 31% and 37% were also found to result in increased hexose yields by 1.3-times to 2.2-times released from enzymatic hydrolysis after NaOH or H_2_SO_4_ pretreatments. The findings indicated that hemicelluloses were the dominant and positive factor, whereas cellulose and lignin had synergistic and negative effects on biomass digestibility.

**Conclusions:**

Using six pairs of *Miscanthus* samples with different cell wall compositions, hemicelluloses were revealed to be the dominant factor that positively determined biomass digestibility after pretreatments with NaOH or H_2_SO_4_ by negatively affecting cellulose crystallinity. The results suggested potential approaches to the genetic modifications of bioenergy crops.

## Background

Plant cell walls are considerable biomass resources of biofuels and other chemicals. Biomass conversion into biofuels involves three major steps: physical and chemical pretreatments for wall polymer disassociation, enzymatic hydrolysis for soluble sugar release, and yeast fermentation for ethanol production [1-4]. Due to plant cell wall recalcitrance, biomass conversion is very expensive [2]. Recalcitrance is mainly determined by the wall polymer features as well as the various interactions among cellulose, hemicelluloses, lignin and pectin [2-4]. The genetic modification of plant cell walls is proposed to be a promising solution for reducing recalcitrance [4,5]. Hence, the effects of wall polymers on biomass digestibility need to be understood.

Cellulose is a major wall polysaccharide accounting for 28%–30% of dry matter in typical forage grasses and 42%–45% in wood [5,6]. Cellulose is a high-molecular-weight linear polymer composed of β-1, 4-glucans [7]. The hydrogen bonds formed between β-1, 4-glucan chains significantly determine cellulose crystallinity [8,9]. Over the past years, the crystallinity index (CrI) has been used to account for cellulose crystallinity by characteristic X-ray diffraction patterns and solid-state ^13^C nuclear magnetic resonance spectra [8,10]. Cellulose crystallinity is reportedly a negative factor that affects biomass hydrolysis [11-13].

Hemicelluloses are polysaccharides containing various monosaccharide subunits [14]. They can be extracted with different concentrations of alkali, acid and other chemicals [15]. It remains unclear about hemicelluloses crosslink with cellulose and lignin, and their effects on cellulose crystallinity and biomass degradation are not well known.

Lignin is composed of three major phenolic components: p-coumaryl alcohol (H), coniferyl alcohol (G), and sinapyl alcohol (S) [16]. An association exists between lignin and biomass recalcitrance [17,18]. The efficiency of biomass saccharification during biofuel production is strongly affected not only by the total amount of lignin but also by the lignin monomer composition in plants [19-21]. The phenolic acid-based interconnections between polysaccharides and lignin also influence biomass digestibility [22].

*Miscanthus* is a C4 perennial plant that has the highest biomass yield among grassy plants, and is currently considered as the leading candidate for biofuel feedstock [23-25]. Given the good adaptability of *Miscanthus* to various environmental conditions, we collected more than 1400 natural *Miscanthus* accessions nationwide and determined 200 typical samples that represented diverse cell wall compositions [25]. In the present study, we selected 12 representative *Miscanthus* samples and then analyzed the biomass saccharification after pretreatments of NaOH and H_2_SO_4_ with different concentrations and sequential enzymatic hydrolysis. Subsequently, we characterized the different effects of three major wall polymers on biomass digestibility in *Miscanthus*.

## Results

### *Miscanthus* cell wall composition and lignocellulose crystallinity

Twelve *Miscanthus* samples were divided into six pairs that each possessed a different cell wall composition, including cellulose, hemicelluloses, and lignin (Table 1). The first three pairs (I, II, and III) mainly showed the different (>25%) of single wall polymer (hemicelluloses, cellulose, lignin), whereas two wall polymers changed in the last three pairs (IV, V, and VI). Despite that hemicelluloses in pairs III and VI were significantly altered by t-test (*p* < 0.05 or 0.01, n = 3), their varied rates were 15% and −10% respectively, which were much lower than the lignin rate (−31%) in Pair III and cellulose (−31%) and lignin (−37%) in Pair VI. Meanwhile, the standard error of the CrI method was detected at ±0.05 ~ 0.15 (n = 5), indicating that each pair of samples had significantly different CrI values.

**Table 1:** **Cell wall composition and lignocellulose crystalline index in*Miscanthus*samples**

**Pair**	**Sample**	**Cell wall composition (% Dry matter)**						**CrI**^**&**^	
		**Cellulose**		**Hemicelluloses**		**Lignin**		**Raw material**	
**I**	Mlu26 (H) ^§^	29.86 ± 1.47	5%^**@**^	25.84 ± 0.73**	**25%**	25.10 ± 0.11	−4%	39.15	−28%
	Msi34 (L)	28.30 ± 0.58		**20.04 ± 0.44**		26.19 ± 0.41		51.77	
**II**	Mfl03 (H)	26.85 ± 0.71**	**−29%**	19.57 ± 0.27	−3%	24.91 ± 1.40	−6%	46.26	−19%
	Mlu01 (L)	**35.88 ± 1.62**		20.09 ± 0.42		26.36 ± 0.49		55.9	
**III**	Mfl40 (H)	30.66 ± 0.55	−7%	22.31 ± 0.16**	15%	21.99 ± 0.30**	**−31%**	45.79	−27%
	Msa02 (L)	32.96 ± 2.52		19.28 ± 0.46		**29.95 ± 0.63**		59.83	
**IV**	Msi56 (H)	36.70 ± 0.64**	**35%**	27.00 ± 0.57**	**39%**	24.40 ± 0.33	2%	38.92	−21%
	Mfl04 (L)	**25.81 ± 0.63**		**18.12 ± 0.20**		23.89 ± 0.52		47.86	
**V**	Msa20 (H)	27.10 ± 1.22	−1%	24.70 ± 0.32**	**25%**	27.38 ± 0.39**	**22%**	46.13	4%
	Mfl08 (L)	27.27 ± 0.44		**19.12 ± 0.11**		**21.90 ± 0.54**		44.48	
**VI**	Mfl27 (H)	27.86 ± 0.70**	**−31%**	19.22 ± 0.65*	−10%	20.49 ± 0.64**	**−37%**	33.84	−31%
	Mlu12 (L)	**38.07 ± 0.37**		21.16 ± 0.02		**29.80 ± 0.29**		46.29	

* and ** A significant difference at pair by *t*-test at *p* < 0.05 and 0.01 (n = 3); @ Percentage of the increased or decreased level at pair: subtraction of two samples divided by means of two values at pair; & CrI method was detected at ±0.05 ~ 0.15 (n = 5); § (H) or (L) Indicated the sample in the pair with high (H) or low (L) biomass digestibility.

In general, the wall polymer alteration could lead to a different cellulose CrI. The Mlu26 sample (Pair I) showed the increased hemicelluloses level by 25% compared with Msi34, resulting in the reduced cellulose CrI by 28%. By contrast, Mlu01 (pair II) contained significantly higher cellulose content by 29% than Mfl03, leading to the increased CrI by 19%. Similarly, the increased lignin level in Msa02 (pair III) resulted in a much higher CrI value by 27% than Mfl40.

While the hemicelluloses level increased in the samples with high cellulose or lignin content (Msi56 or Msa20), the cellulose CrI values were relatively lower by 21% in pair IV or with little change by less than 5% in pair V compared with its paired sample Mfl04 or Mfl08. In comparison, although both cellulose and lignin contents remained much higher in Mlu12 than in Mfl27 in pair VI, the cellulose CrI increased by 31%. This value was the largest increase rate among the six pairs.

### Positive effect of the hemicellulose level on biomass digestibility

Biomass digestibility was defined by accounting for either the sugar yield (hexoses/cellulose) released from hydrolysis by a crude cellulase mixture of lignocellulose after chemical pretreatment, or the total sugar yield (hexoses and pentoses/dry weight) from both pretreatment and enzymatic hydrolysis. In this study, three concentrations of NaOH and H_2_SO_4_ (0.25% or 0.5%, 1%, and 4%) were used for chemical pretreatments, and commercial crude mixed cellulases were used for enzymatic hydrolysis (Additional file 1: Tables S1 and S2).

**Figure 1:**
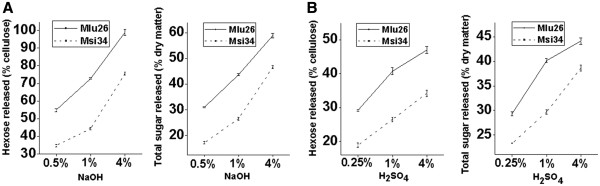
**Hemicelluloses positive effect on Biomass digestibility.***Miscanthus* sample with a relatively higher hemicelluloses level (Mlu26) showing an enhanced biomass digestibility after pretreatments with (**A**) NaOH or (**B**) H_2_SO_4_ at three concentrations. The biomass digestibility was accounted by either hexoses yield (% cellulose) released from enzymatic hydrolysis after pretreatment or total sugar yield (% dry matter) released from both pretreatment and enzymatic hydrolysis.

With respective to its relatively high hemicellulose level (Table 1), the Mlu26 sample (Pair I) was found to have significantly higher biomass digestibility than Msi34 after pretreatment with three concentrations of NaOH or H_2_SO_4_ by *t*-test (n = 3) (Figure 1). Particularly, after 4% NaOH pretreatment, Mlu26 displayed an extremely high hexose yield at 99% cellulose, whereas Msi34 remained less than 76% (Figure 1A and Additional file 1: Table S1). Both samples showed much more effective biomass saccharification rates (hexose/cellulose) after NaOH pretreatments than after H_2_SO_4_ pretreatments (Figure 1B and Additional file 1: Table S1). Despite of the relatively higher hemicellulose level of Mlu26 than Msi34, both samples showed very similar monosaccharide compositions (Table 2), indicating a typical xylan structure of grassy plant that is mainly composed of xylose and arabinose [14]. Hence, increasing the total hemicellulose level without altering the hemicellulose monosaccharide composition can result in significantly enhanced biomass digestibility in *Miscanthus*.

**Table 2:** Monosaccharide composition of hemicelluloses (% of total)

**Pair**	**Sample**	**Rha**	**Fuc**	**Ara**	**Xyl**	**Man**	**Glu**	**Gal**
**I**	Mlu26 (H)	0.20%*	0.01%	11.10%	86.15%	0.21%	1.14%	1.20%
	Msi34 (L)	0.21%	ND	11.17%	86.01%	0.10%	1.16%	1.34%

* Percentage of total monosaccharides; ND, non-detectable.

### Negative effects of cellulose and lignin contents on biomass saccharification

**Figure 2:**
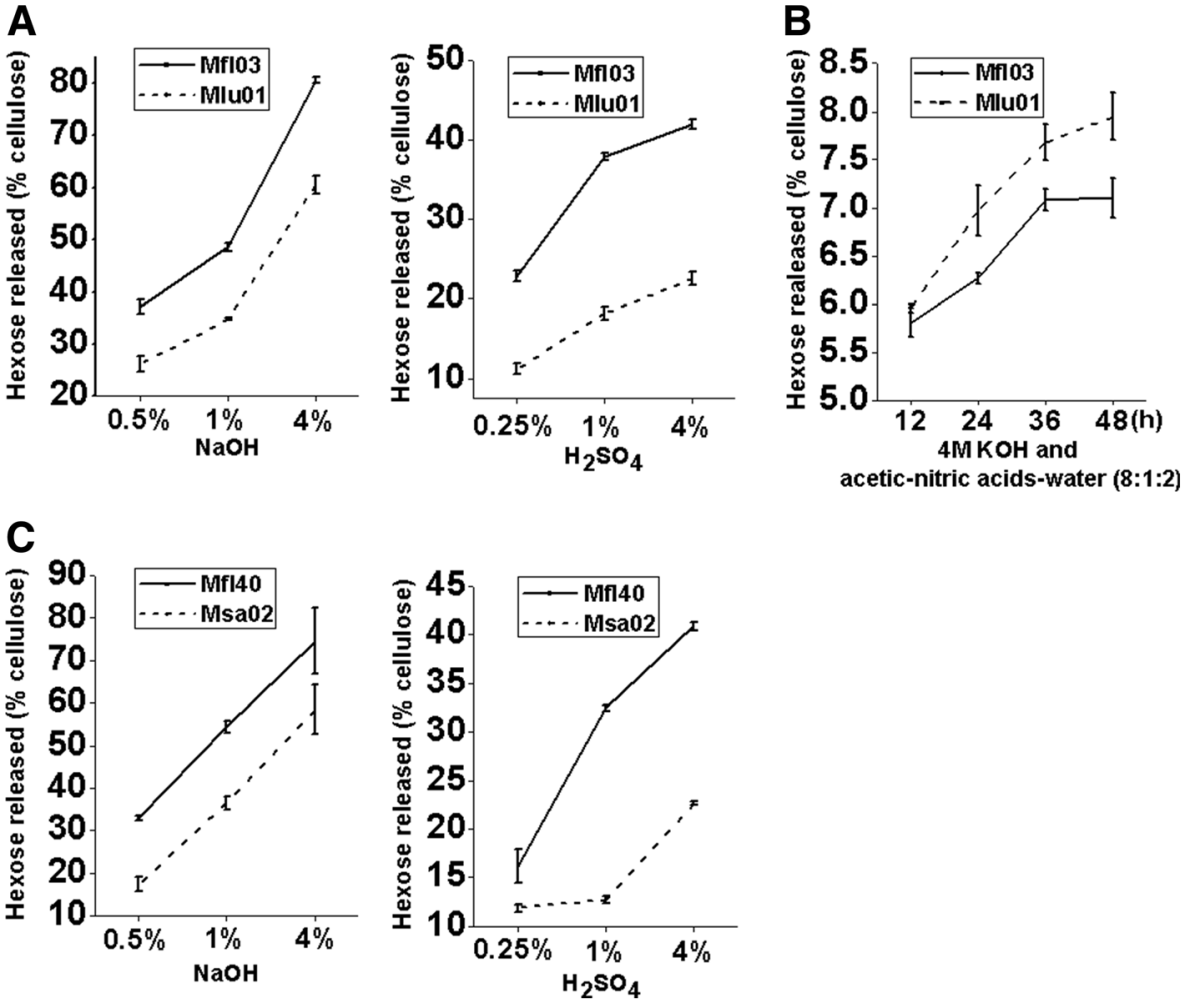
**Cellulose and lignin negative effects on biomass digestibility.***Miscanthus* sample (**A**) with relatively higher cellulose level (Mlu01) or (**C**) lignin content (Msa02) showing a decreased biomass digestibility after pretreatments with NaOH or H_2_SO_4_ at three concentrations; (**B**) Glucose yield released by enzymatic hydrolysis of crystalline cellulose obtained from 4 M KOH and acetic-nitric acids extractions of biomass samples (Methods: plant cell wall fractionation).

In terms of relatively higher cellulose content (Table 1), Mlu01 (pair II) showed remarkably lower biomass digestibility than Mfl03 after pretreatments with three concentrations of NaOH or H_2_SO_4_ and sequential enzymatic hydrolysis by *t*-test (Figure 2, Additional file 1: Table S1 and Table S2). However, the two samples displayed very different hexose yields under H_2_SO_4_ and NaOH pretreatments (Figure 2A). For instance, Mlu01 had a hexose yield 1.3-times to 1.4-times less than Mfl03 after pretreatments with three concentrations of NaOH, but 1.7-times to 2.1-times after H_2_SO_4_ pretreatments (Additional file 1: Table S1). But, both samples in pair II exhibited extremely low enzymatic hydrolysis rates of lignocellulose after pretreatment with 4 M KOH followed by acetic-nitric acids-water (8:1:2), and Mlu01 even displayed a significantly higher hexoses yield than Mfl03 did by *t*-test (Figure 2B).

With regard to its high lignin level (Table 1), Msa02 (pair III) displayed much lower biomass digestibility than Mfl40 (Figure 2C, Additional file 1: Tables S1 and S2). The two samples also showed very different hexose yields (2.6- and 1.8-times) after 1% and 4% H_2_SO_4_ pretreatments, compared with those after 1% and 4% NaOH pretreatments (1.5-times and 1.3-times) (Additional file 1: Table S1). The two samples in pair III were found to have quite different lignin monomer compositions, particularly the proportions of S and H monomers (Table 3). Also, the S/G ratio of Mfl40 was 0.53, but that of Msa02 was 0.82, suggesting the negative effect of a high S/G ratio on biomass saccharification in *Miscanthus*.

**Table 3:** Monomer composition of lignin (% of total)

**Pair**	**Sample**	**H**	**G**	**S**	**S/G**	**H/G**	**S/H**
**III**	Mfl40 (H)	33.65%*	43.28%	23.06%	0.53	0.78	0.69
	Msa02 (L)	19.69%	44.03%	36.28%	0.82	0.45	1.84

* Percentage of total monomers.

### Dominant effects of hemicelluloses on biomass digestion

**Figure 3:**
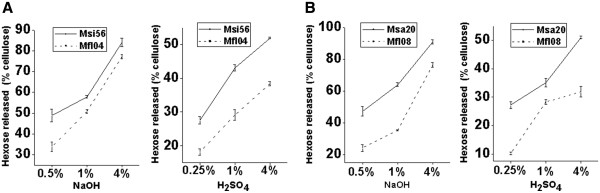
**Hemicelluloses dominant effects on biomass saccharification.** Increase of hemicelluloses level in (**A**) cellulose-high sample (Msi56) or (**B**) lignin-rich sample (Msa20) resulting in a raised biomass digestibility after pretreatments with NaOH or H_2_SO_4_ at three concentrations.

As aforementioned, the high cellulose and lignin levels of the pair II and III samples significantly resulted in low biomass digestibility (Figure 2). However, increasing the hemicelluloses content of the pair IV or V samples, which had high cellulose (Msi56) or lignin (Msa20) levels (Table 1), can result in biomass digestion at high efficiency compared with their paired samples (Mfl04 and Mfl08) (Figure 3, Additional file 1: Tables S1 and Table S2). This result suggested the dominant role of hemicelluloses in biomass digestibility despite of increased cellulose or lignin contents. The hemicelluloses monosaccharide compositions were also not significantly altered in pairs IV and V (Additional file 1: Table S3 and Table S4). In addition, in terms of the increased hemicelluloses level in pair V, Msa20 had a lower S/G ratio (0.43) than Mfl08 (0.68) (Table 4), different from pair III in which Msa02 had a high lignin level and a high S/G ratio.

**Table 4:** Monomer composition of lignin (% of total)

**Pair**	**Sample**	**H**	**G**	**S**	**S/G**	**H/G**	**S/H**
**V**	Msa20 (H)	24.09%*	52.92%	22.99%	0.43	0.46	0.95
	Mfl08 (L)	35.84%	38.29%	25.87%	0.68	0.94	0.72

* Percentage of total monomers.

### Synergistic effect of cellulose and lignin levels on lignocellulose digestibility

**Figure 4:**
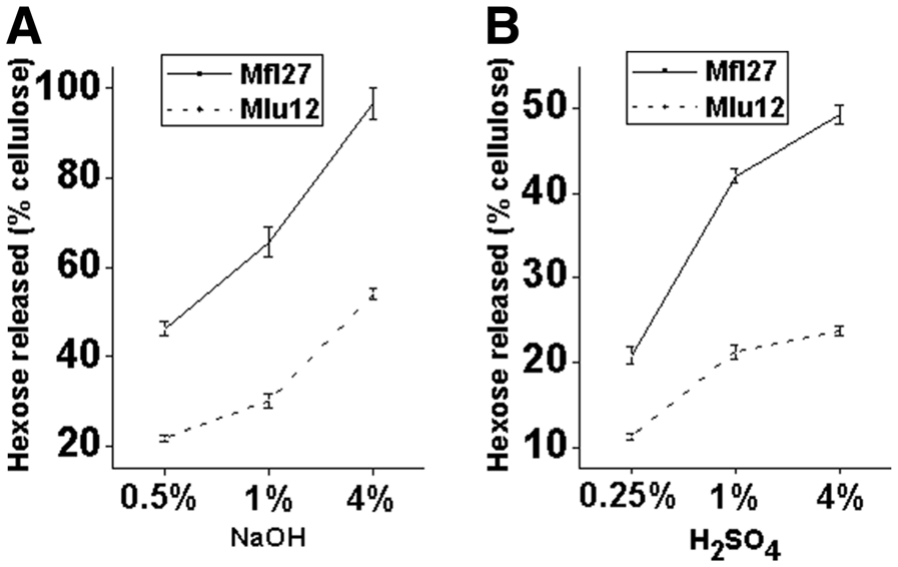
**Synergistic effects of cellulose and lignin on biomass saccharification.** Increase of both cellulose and lignin level (Mlu12) leading to a much decreased biomass digestibility after pretreatments with NaOH (**A**) or H_2_SO_4_ (**B**) at three concentrations.

Although the increase in cellulose or lignin levels in pair II or III resulted in reduced biomass digestion at different rates under NaOH and H_2_SO_4_ pretreatments (Figure 2), increasing both the cellulose and lignin levels in pair VI can result in extremely reduced biomass saccharification at similar efficiencies under NaOH and H_2_SO_4_ pretreatments (Figure 4). For instance, compared with Mfl27, Mlu12 in pair VI showed reduced hexoses yield by 1.8-times to 2.2-times after NaOH pretreatments or 1.9-times to 2.0-times after H_2_SO_4_ pretreatments (Additional file 1: Table S1). This finding suggested a synergistic effect of cellulose and lignin on biomass digestibility. Accordingly, we observed that the two samples in pair VI had different cell wall structures including hemicelluloses monosaccharide composition (Additional file 1: Table S5), lignin monomer constitution (Additional file 1: Table S6), and phenol-linkage types (Table 5 and Additional file 1: Table S7). Hence, Mlu12 displayed a higher ratio than Mfl27 either on Xyl/Ara in hemicelluloses or S/G in lignin, as well as more linked phenols, suggesting a wall polymer interlinked network evident in the two samples in pair VI.

**Table 5:** Linked Phenols of Mfl27 and Mlu12 (μmol/g Dry Matter)

**Linkage**	**Sample**	**H-**	**G-**	**S-**	**AV-**	**AS-**	**PCA-**	**FA-**	**Total**
**Ester- and ether-**	Mfl27 (H)	3.00 (3.67%)*	11.56 (14.16%)	9.30 (11.40%)	6.00 (0.74%)	13.77 (16.87%)	8.56 (10.49%)	29.41 (36.05%)	81.58
	Mlu12 (L)	3.85 (3.71%)	20.44 (19.68%)	30.16 (29.04%)	6.04 (0.58%)	12.62 (12.15%)	7.40 (7.12%)	23.36 (22.49%)	103.88

H-: *p*-Hydroxybenzaldehyde, G-: Vanillin, S-: Syringaldehyde, AV-: Acetovanillone, AS-: Acetosyringone, PCA-: *p-*Coumaric acid, FA-: Ferulic acid, SA-: Sinapic acid;

* percentage of total linked-phenols.

### Scanning electron microscopic observation

**Figure 5:**
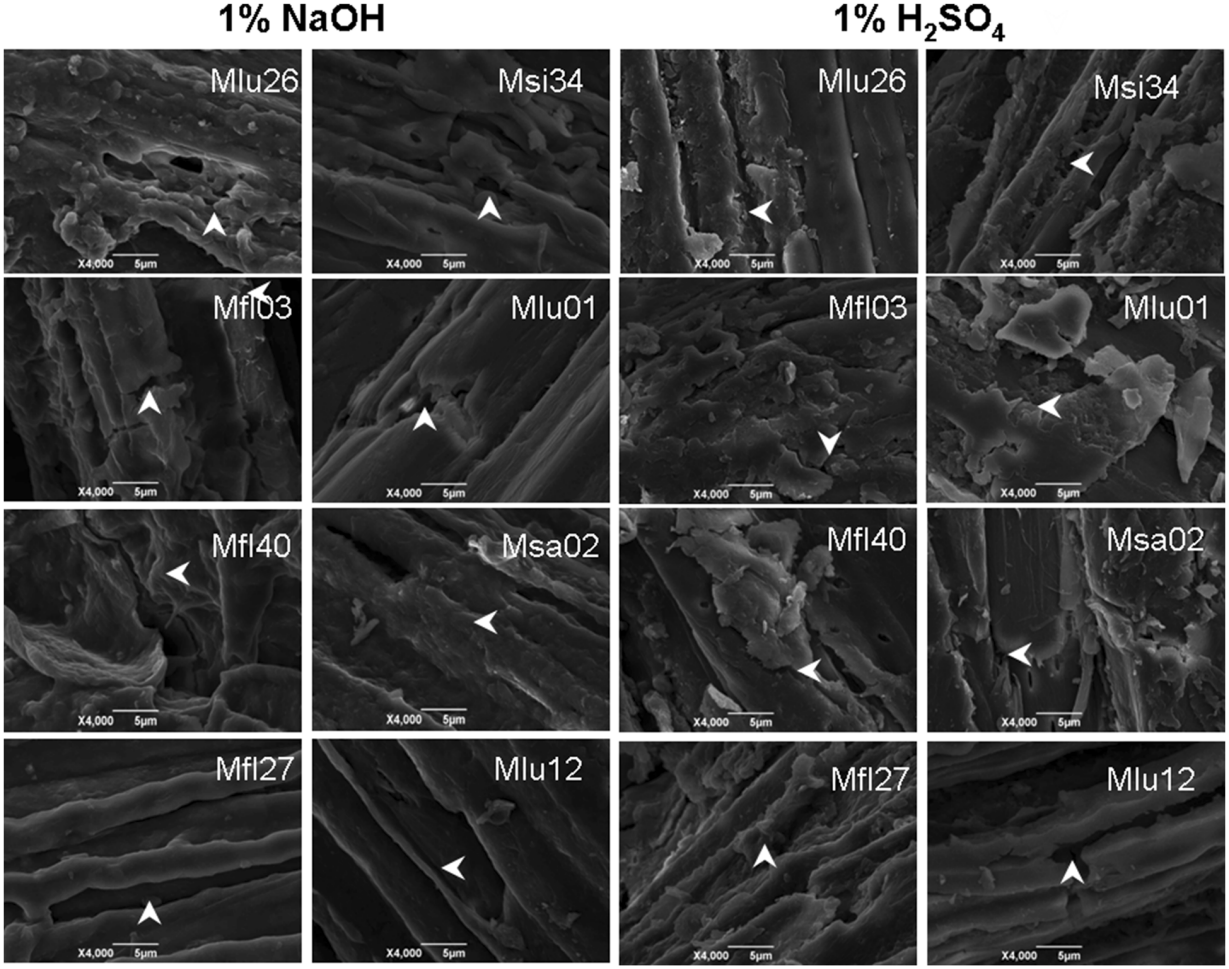
**SEM imagines of biomass residues obtained from pretreatment with 1% NaOH or 1% H**_**2**_**SO**_**4**_**, and sequential enzymatic hydrolysis.** Sample (Mlu26, Mfl03, Mfl40, Mfl27) with a relatively higher biomass digestibility showing a coarse surface indicated as arrow, and sample (Msi34, Mlu01, Msa02, Mlu12) displaying a flat face.

The residues of the samples in four pairs after pretreatments with 1% NaOH or 1% H_2_SO_4_ after sequential enzymatic hydrolysis were visualized under a scanning electron microscope (Figure 5). The samples (Mlu26, Mfl03, Mfl40, and Mfl27) that had higher biomass digestibility displayed a coarse surface of biomass residue, whereas their paired samples (Msi34, Mlu01, Msa02, and Mlu12) exhibited a relatively smooth face. Particularly, all samples remained more small grained on the surface after H_2_SO_4_ pretreatments compared with NaOH pretreatments. This result suggested that biomass was not well extracted with H_2_SO_4_, and the remaining small grains may affect cellulase enzyme penetration and accessibility into the cellulose surface.

### Correlation between lignocellulose crystallinity and biomass digestibility

**Figure 6:**
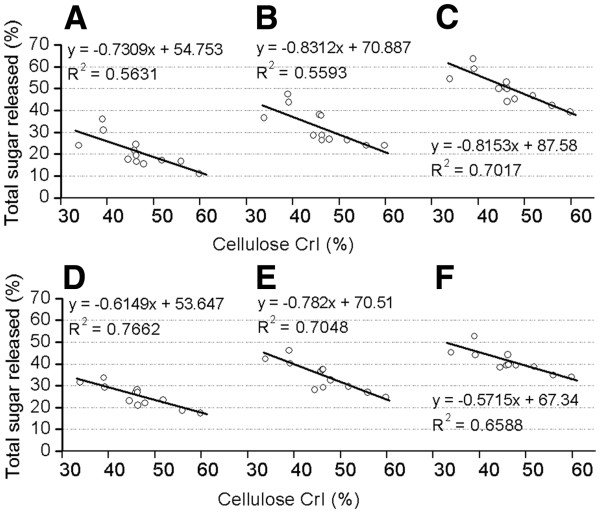
**Correlation between cellulose CrI and total sugar yield.** Total sugar yield released from both enzymatic hydrolysis and pretreatment with (**A**) 0.5% NaOH, (**B**) 1% NaOH, (**C**) 4% NaOH, (**D**) 0.25% H_2_SO_4_, (**E**) 1% H_2_SO_4_, (**F**) 4% H_2_SO_4_.

**Figure 7:**
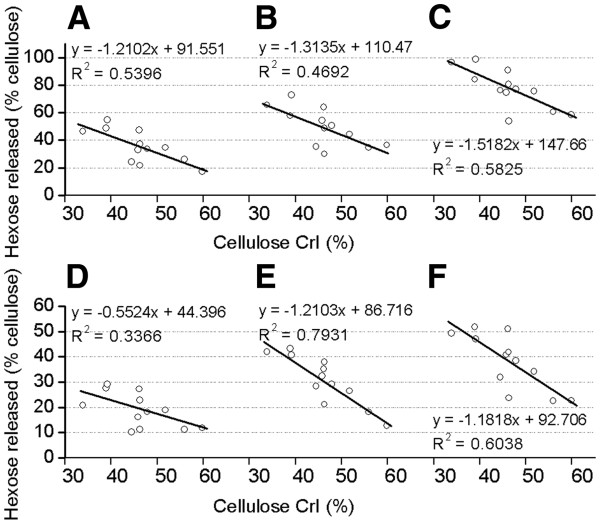
**Correlation between cellulose CrI and hexoses yield.** Hexoses yield released from enzymatic hydrolysis after pretreatment with (**A**) 0.5% NaOH, (**B**) 1% NaOH, (**C**) 4% NaOH, (**D**) 0.25% H_2_SO_4_, (**E**) 1% H_2_SO_4_, (**F**) 4% H_2_SO_4_.

A correlation was calculated to account for the relationship between lignicellulose crystallinity (CrI) and biomass digestibility among the twelve samples after pretreatments with three concentrations of NaOH or H_2_SO_4_ (Figures 6 and 7). Significantly, a negative correlation was observed with *R*^2^ > 0.70 values for the total sugar yield released after 4% NaOH or 0.25% H_2_SO_4_ pretreatments, and for the hexoses yield after 1% H_2_SO_4_ pretreatment. The negative correlation coefficient values were calculated to range from 0.73 to 0.89 at *p* < 0.01 (n = 3) for almost all pretreatments, except 0.25% H_2_SO_4_ and 1% NaOH with 0.58 and 0.685 values at *p* < 0.05 (Additional file 1: Tables S8 and S9). Therefore, this study confirmed that lignocellulose crystallinity (CrI) was a significant negative parameter that affected biomass digestibility despite the different cell wall compositions of the six pairs of samples.

## Discussion

*Miscanthus* is considered as a promising bioenergy crop. However, plant cell wall recalcitrance determines its cost-effective conversion into biofuels. Considering that the genetic modification of plant cell walls is proposed to reduce recalcitrance, the crucial factor in wall polymers that affects biomass digestibility needs to be identified [25]. Due to the complicated structures and diverse functions of plant cell walls [25-27], the effects of the three major wall polymers (cellulose, hemicelluloses, and lignin) on biomass digestion were initially compared. Hence, this study focused on the analysis of six pairs of *Miscanthus* samples that had different cell wall compositions.

Studies on the effects of the three major wall polymers, particularly cellulose and hemicelluloses, on lignocellulose digestibility in plants are very limited. The effective hydrolysis of hemicelluloses due to the soluble and extractable properties of these polymers has been described [28]. In the current work, total hemicelluloses level was demonstrated to be the positive and dominant factor that affected the high biomass saccharification efficiency due to its negative effect on lignocellulose crystallinity (CrI). Hemicelluloses are proposed to deposit into cell walls via crosslink to cellulose by hydrogen bonds [14,29]. Thus, hemicelluloses can reduce cellulose crystallization and the negative effect of the hemicelluloses level may not depend on the cellulose as well as lignin levels (Table 1).

Cellulose crystallinity (CrI) reportedly affects biomass digestibility negatively because the reduction of cellulose CrI may result in efficient cellulase enzyme penetration and high affinity to cellulose substrate [30,31]. However, little is known about the impact of the three wall polymers on cellulose crystallinity. Apart from the abovementioned negative hemicelluloses effect, the cellulose and lignin levels were found to be positive factors (Table 1). The positive effect of cellulose levels may be due to the relatively low hemicelluloses proportion or smaller non-crystalline cellulose region. With respect to the positive effect of lignin, lignin was assumed to interact with hemicelluloses rather than with cellulose, which may indirectly reduce hemicelluloses cross-linking to cellulose. This assumption also confirmed that increased cellulose and lignin levels can lead to increased cellulose CrI at higher rates as observed in the pair VI samples (Table 1). Although the S/G ratio in lignin is recently reported to be a dual factor that affects biomass digestibility [20,32], the mechanism remains unknown. In the present study, *Miscanthus* samples with high S/G ratios were found to have relatively higher cellulose CrI values, which suggested that S monomer may have a different interlinking with wall polymers.

Acid and alkali chemicals such as H_2_SO_4_ and NaOH are extensively used in biomass pretreatments. However, the two chemicals are found to have different mechanisms for biomass depolymerization [33]. Alkali pretreatment can mostly cause the dissociation of entire wall polymers by breaking hydrogen and other covalent bonds, whereas acid pretreatment induces the partial release of monosaccharides, oligosaccharides, and lignin monomers by splitting strong chemical bonds under high temperature [34-36]. Hence, smaller residues remained on the cellulose surface after 1% H_2_SO_4_ pretreatment than after 1% NaOH pretreatment (Figure 5), leading to a relatively lower biomass saccharification rate after 1% H_2_SO_4_ pretreatment (Additional file 1: Table S1). This result also indicated that increasing the cellulose or lignin levels of pairs II and III can result in much lower hexose yields after H_2_SO_4_ pretreatments than after NaOH (Figure 2). In other words, the result confirmed that increasing the hemicellulose level of pair I or decreasing the cellulose and lignin levels of pair VI (Table 1) can lead to increased biomass digestibility at similar rates (1.3-times to 1.5-times for pair I and 1.8-times to 2.2-times for pair IV) between H_2_SO_4_ and NaOH pretreatments (Additional file 1: Table S1).

Plant cell wall mutants have been used to account for wall polymers and biomass digestibility. Generally, most mutants show growth-defective and biomass-reduced phenotypes [37], these mutants can not be directly used as energy crops for biofuel purposes. In particular, multiple alterations in cell wall compositions and structures render some mutants not even worthy of consideration as experimental materials. Based on a rich natural germplasm resource, we selected six pairs of *Miscanthus* samples with different cell wall compositions. These samples were able to demonstrate the effect of each wall polymer on biomass saccharification, and can thus be used as genetic materials for energy crop breeding. Thus, this study provides a fundamental strategy for the genetic engineering of plant cell walls toward bioenergy crop selection.

## Conclusion

Based on the analysis of six typical pairs of *Miscanthus* samples, hemicelluloses were demonstrated to be a positive and dominant factor that affects biomass digestibility. By contrast, cellulose and lignin played synergistic and negative effects on the sugar yields generated by the enzymatic hydrolysis of biomass after chemical pretreatments. Correlation analysis confirmed that cellulose CrI was the parameter that can account for biomass saccharification efficiency. Cellulose CrI can also be negatively affected by hemicelluloses, but positively affected by cellulose and lignin. Increased hemicelluloses level or decreased cellulose and lignin contents can lead to enhanced biomass digestibility with similar rates under H_2_SO_4_ and NaOH pretreatments. Hence, the proposed approach has potential application in the genetic engineering of bioenergy crops.

## Methods

### Plant samples

The *Miscanthus* samples were typically selected from wild *Miscanthus* germplasm accessions collected in China in 2007. The samples harvested from Hunan experimental field in 2009 season were dried at 50°C after treated at 105°C for 5 min. The dried tissues were ground through a 40 mesh sieve and stored in a dry container until use.

### Plant cell wall fractionation

The polysaccharides were extracted as the method from Peng et al. with minor modification [15]. The crude cell wall material was suspended in 0.5% (w/v) ammonium oxalate and heated for 1 h in a boiling water bath, and the supernatants were combined as total pectin. The remaining pellet was suspended in 4 M KOH containing 1.0 mg mL^-1^ sodium borohydride for 1 h at 25°C, and the combined supernatant was neutralized, dialyzed and lyophilized as hemicelluloses. The KOH non-extractable residue was further extracted with acetic-nitric acids for 1 h at 100°C and the remaining pellet was defined as crystalline cellulose. All samples were carried out in triplicate for wall fractionations.

### Colorimetric assay of hexoses and pentoses

UV/VIS Spectrometer (Shanghai MAPADA Instruments Co., Ltd. V-1100D) was used for the absorbance measurement [27]. D-Glucose, D-xylose, ferric chloride and orcinol were purchased from Sinopharm Chemical Reagent Co., Ltd. Anthrone was obtained from Sigma-Aldrich Co. LLC. Total hexoses assay: 1.0 mL aqueous sample (containing 20–100 μg hexoses) was added to 0.2% anthrone (2.0 mL) in conc H_2_SO_4_, mixed well and incubated in a boiling water bath for 5 min [38]. After the sample was cooled, its absorbance was read at 620 nm. For the determinations of cellulose, the cellulose was dissolved in 67% (v/v) H_2_SO_4_ (1.0 mL) with shaking at 25°C for 1 h, and then 10.0 μL aliquot was used for the anthrone/H_2_SO_4_ method. The anthrone/H_2_SO_4_ assay was used to determine cellulose content and hexoses yield released from pretreatment and enzymatic hydrolysis. Total pentoses assay: 1.0 mL aqueous sample (containing 5–40 μg pentoses ) was added to 6% orcinol (134 μL) in ethanol, followed by 0.1% FeC1_3_·6H_2_O (2.0 mL) in conc HCl, then mixed well and incubated in a boiling water bath for 20 min. After it was cooled, the sample was mixed again and its absorbance was read at 660 nm [38]. Both anthrone/H_2_SO_4_ and orcinol/HCl methods were used to measure total hemicelluloses levels. Because the high pentoses level in the sample can affect the absorbance reading at 620 nm for hexoses content by anthrone/H_2_SO_4_ method, the deduction from pentoses reading at 660 nm was carried out for final calculation of hexoses level. All experiments were carried out in biological triplicate

### Hemicelluloses monosaccharide determination by GC-MS

TFA and *myo*-inositol were purchased from Aladdin Reagent Inc. Acetic anhydride and acetic acid were obtained from Sinopharm Chemical Reagent Co., Ltd. 1-methylimidazole was purchased from Sigma-Aldrich Co. LLC. Monosaccharide standards including L-rhamnose, L-arabinose, L-fucose, D-xylose, D-galactose, D-glucose and D-mannose, were obtained from Sinopham Chemical Reagent Co., Ltd.

Acid hydrolysis: The combined supernatants from 4 M KOH fraction were dialyzed for 36 h after neutralization with acetic acid. The polysaccharides dissolved in 2.5 mL TFA (2 M) were heated in a sealed tube at 121°C in an autoclave (15 psi) for 1 h. *Myo*-inositol (200 μg) was added as the internal standard. The supernatant was dried under vacuum at 38°C to remove TFA.

Derivatisation of monosaccharides to alditoal acetates: Distilled water (800 μL) and a freshly prepared solution of NaBH_4_ (400 μL, 100 mg/mL in 6.5 M aqueous NH_3_) were added to each sample. Sample was capped, mixed well and incubated at 40°C for 30 min. Excess NaBH_4_ was decomposed by adding acetic acid (800 μL). 400 μL Sample was then moved into a 25 mL glass tube. Acetic anhydride (4 mL) was added to the tube and the solution mixed again. Then 1-methylimidazole (600 μL) was added. After mixing, the sample was allowed to stand for 10 min. Excess acetic anhydride was decomposed by adding distilled water (10 mL). Then dichloromethane (3 ml) was added, mixed gently, centrifuged (2,000 *g*, 10 seconds) for phase separation. After removing the upper phase, the sample was washed with distilled water (3 × 20.0 mL). The collected lower phase was dehydrated by adding with anhydrous sodium sulfate and stored at −20°C until analyzed by GC-MS (SHIMADZU GCMS-QP2010 Plus).

GC-MS Analytical Conditions: Restek Rxi-5 ms, 30 m × 0.25 mm ID × 0.25um df column. Carrier gas: He. Injection Method: Split. Injection port: 250°C, Interface: 250°C. Injection Volume: 1.0 μL. The temperature program: from 170°C (held for12 min) to 220°C (held for 8 min) at 3°C/min. Ion source temperature: 200°C, ACQ Mode: SIM. The mass spectrometer was operated in the EI mode with ionization energy of 70 ev. Mass spectra were acquired with full scans based on the temperature program from 50 to 500 m/z in 0.45 s. Calibration curves of all analytes routinely yielded correlation coefficients 0.999 or better.

### Total lignin assay

Total lignin content was determined by two-step acid hydrolysis method according to Laboratory Analytical Procedure of the National Renewable Energy Laboratory. The lignin includes acid-insoluble and -soluble lignin. The acid-insoluble lignin was calculated gravimetrically as acid-insoluble residue after correction for ash, and the acid-soluble lignin was measured by UV spectroscopy.

Acid-insoluble lignin determination: 0.5 g sample recorded as W1. Each sample was run in triplicate. The sample was extracted with benzene-ethanol (2:1, v/v) in a Soxhlet for 4 h, and then air-dried in hood overnight. The sample was hydrolyzed with 10 mL 72% H_2_SO_4_ (v/v) in shaker at 30°C for 1.5 h. After hydrolysis, the acid was diluted to a concentration of 2.88%, and then placed in the autoclave for 1 h at 121°C (15 psi). The autoclaved hydrolysis solution was vacuum-filtered through the previously weighed filtering crucible. The filtrate was captured in a filtering flask for acid-soluble lignin. The lignin was washed free of acid with hot distilled water and the crucible and acid-insoluble residue was dried in an oven at 80°C until constant weight was achieved. Then, the samples were removed from the oven and cool in a dry-container. The weight of the crucible and dry residue was recorded to the nearest 0.1 mg (W2). At last the dried residue was ashed in the muffle furnace at 200°C for 30 min and 575°C for 4 h. The crucibles and ash were weighed to the nearest 0.1 mg and recorded the weight (W3). Acid-insoluble lignin (AIL) on original sample was calculated as the following: AIL (%) = (W2-W3) × 100/W1%.

Acid-soluble lignin determination: The acid-soluble lignin was solubilized during the hydrolysis process, and was measured by UV spectroscopy. The hydrolysis liquor obtained previously was transfer into 250 mL volumetric flask and brought up to 250 mL with 2.88% sulfuric acid. The absorbance of the sample was read at 205 nm on a UV–vis spectroscopy (Beckman Coulter Inc., Du800), and 2.88% sulfuric acid was used as blank. The method of calculation about the amount of acid soluble lignin was as follows: ASL (%) = (A × D × V/1000 × K × W1) × 100%. A (absorption value), D (Dilution ratio of the sample), K (absorptivity constant) = 110 L/g/cm. Total lignin (%) = ASL% + AIL%. All experiments were carried out in triplicate.

### Lignin monomer detection by HPLC

Standard chemicals: *p*-Hydroxybenzaldehyde(H), vanillin(G) and syringaldehyde (S) were purchased from Sinopharm Chemical Reagent Co., Ltd. The sample was extracted with benzene-ethanol (2:1, v/v) in a Soxhlet for 4 h, and the remaining pellet was collected as cell wall residue (CWR). The procedure of nitrobenzene oxidation of lignin was conducted as follows; 0.05 g CWR was added with 5 mL 2 M NaOH and 0.5 mL nitrobenzene, and a stir bar was put into a 25 mL Teflon gasket in a stainless steel bomb. The bomb was sealed tightly and heated at 170°C (oil bath) for 3.5 h and stirred at 20 rpm. Then, the bomb was cooled with cold water. The chromatographic internal standard (ethyl vanillin) was added to the oxidation mixture. This alkaline oxidation mixture was washed 3 times with 30 mL CH_2_C1_2_/ethyl acetate mixture (1/1, v/v) to remove nitrobenzene and its reduction by-products. The alkaline solution was acidified to pH 3.0-4.0 with 6 M HCl, and then extracted with CH_2_CI_2_/ethyl acetate (3 × 30 mL) to obtain the lignin oxidation products which were in the organic phase. The organic extracts were evaporated to dryness under reduced pressure 40°C. The oxidation products were dissolved in 10 mL chromatographic pure methanol.

HPLC analysis: The solution was filtered with membrane filter (0.22 μm). 20 μL Solution was injected into HPLC (Waters 1525 HPLC) column Kromat Universil C18 (4.6 mm × 250 mm, 5 μm) operating at 28°C with CH_3_OH:H_2_O:HAc (25:74:1, v/v/v) carrier liquid (flow rate: 1.1 mL/min). Calibration curves of all analytes routinely yielded correlation coefficients 0.999 or better, and the detection of the compounds was carried out with a UV-detector at 280 nm.

### Wall-linked phenolics determination by HPLC

Standard chemicals: *trans*-FA and *trans*-*p-*CA, *trans*-Sinapic acid were purchased from Sigma-Aldrich Co. LLC. Acetovanillone (AV) and acetosyringone (AS) were obtained from Biosharp Co., Ltd. The dewaxed CWR (0.2 ± 0.0001 g) was added with 10 mL 1 M NaOH (containing 1.0 mg/mL NaHSO_3_) for 18 h at 30°C in a shaker (150 rpm), centrifuged and washed with distilled water 3 times (3 × 10 mL). The combined supernatant was acidified to pH 2.0 with 6 M HCl, and the acidified solution was extracted with chloroform (3 × 10 mL) after filtration. The combined organic extracts were evaporated to dryness under the reduced pressure at 40°C. The extracts were re-dissolved in 2.0 mL elution phase, prior to HPLC analyses for ester-linked phenolics.

Isolation of total linked phenolics (ester and ether): The de-waxed CWR (0.05 ± 0.0001 g) was added with 10 mL 4 M NaOH (containing 1.0 mg/mL NaHSO_3_) for 2 h at 170°C in a stainless steel bomb with magnetic stirrers (20 rpm). The mixture was acidified to pH 2.0 with 6 M HCl, and the acidified solution was extracted with chloroform (3 × 10 mL) after filtration and then the combined organic extracts were evaporated to dryness under reduced pressure at 40°C. The extracts were re-dissolved in 2.0 mL elution phase, then it was filtered by 0.22 μm membrane and used for HPLC analyses.

HPLC analysis: Separation was performed by HPLC (Waters 1525 HPLC) on a Kromat Universil C18 (4.6 mm × 250 mm, 5 μm) at 28°C. Elution was carried out using a system consisting of solvent with distilled water: methanol: acetic acid (75:24:1, v/v/v) at flow rate: 1.1 mL/min. Quantification of wall-bound phenolics was conducted by external standard method. The amount of ether-linked phenolics was calculated. Calibration curves of all analytes routinely yielded correlation coefficients at 0.999 or better, and the detection of the compounds was carried out with a UV-detector at 280 nm.

### Detection of cellulose crystallinity

X-ray diffraction (XRD) method was used to detect cellulose crystallinity index (CrI) using Rigaku-D/MAX instrument (Uitima III, Japan). The powders of raw biomass samples were laid on the glass sample holder (35 × 50 × 5 mm) and were analyzed under plateau conditions. Ni-filtered Cu Kα radiation (λ = 0.154056 nm) generated at voltage of 40 kV and current of 18 mA, and scanned at speed of 0.0197° /s from 10° to 45°. The crystallinity index (CrI) was estimated using the intensity of the 200 peak (I_200_, θ = 22.5°) and the intensity at the minimum between the 200 and 110 peaks (I_am_, θ = 18.5°) as the follow: CrI = 100 × (I_200_–I_am_)/I_200_. I_200_ represents both crystalline and amorphous materials while Iam represents amorphous material. Standard error of the CrI method was detected at ±0.05 ~ 0.15 using five representative samples in triplicate.

### Scanning electron microscopic (SEM) observation

The biomass residues were collected after pretreatment with NaOH or H_2_SO_4_ and the sequential enzymatic hydrolysis. The samples were washed with distill water, dried under air, and sputter-coated with gold in a JFC-1600 ion sputter (Mito City, Japan). The surface morphology of and treated samples was sputter-coated with gold and observed by scanning electron microscope (SEM JSM-6390/LV, Hitachi, Tokyo, Japan).

### Biomass pretreatment

H_2_SO_4_ pretreatment: The biomass samples (0.5 g) were added with 10 mL H_2_SO_4_ at three concentrations (0.25%, 1%, 4%, v/v), respectively. The tube was sealed and heated at 121°C for 20 min in autoclave (15 psi) after mixing well. Then, the tube was shaken at 150 rpm for 2 h at 50°C, and centrifuged at 3,000 *g* for 5 min. The pellet was washed three times with 10 mL distilled water, and stored at −20°C for enzymatic hydrolysis. All supernatants were collected for determination of total sugars (pentoses and hexoses) released from acid pretreatment, and samples with 10 mL distilled water were shaken for 2 h at 50°C as the control [27]. All samples were carried out in triplicate.

NaOH pretreatment: The biomass sample (0.5 g) was added with 10 mL NaOH at three concentrations (0.5%, 1%, 4%, w/v). The tube was shaken at 150 rpm for 2 h at 50°C, and centrifuged at 3,000 *g* for 5 min. The pellet was washed three times with 10 mL distilled water. All supernatants were collected for determination of total sugars released from alkali pretreatment, and samples with 10 mL distilled water were shaken for 2 h at 50°C as the control. All samples were carried out in triplicate.

### Enzymatic hydrolysis

The remaining residues from various pretreatments were washed 2 times with 10 mL distilled water, and once with 10 mL mixed-cellulases reaction buffer (0.2 M acetic acid-sodium acetate, pH 4.8). The washed residues were added with 10 mL(2 g/L) mixed-cellulases (containing β-glucanase ≥ 6 × 10^4^ U) and cellulase ≥ 600 U and xylanase ≥ 10 × 10^4^ U from Imperial Jade Bio-technology Co., Ltd) at 0.16% (w/w) concentration for H_2_SO_4_ and NaOH pretreated samples. During the enzymatic hydrolysis, the samples were shaking under 150 rpm at 50°C for 48 h. After centrifugation at 3,000 *g* for 10 min, the supernatants were collected for determining amounts of pentoses and hexoses released from enzymatic hydrolysis. The samples with 10 mL reaction buffer were shaken for 48 h at 50°C as the control. All samples were carried out in triplicate.

## Competing interests

The authors declare that they have no competing interests.

## Authors’ contributions

NX and WZ completed major experiments and analyzed the data. SR, FL and CZ, JH participated hemicelluloses composition and lignin monomer determination. HL, QL YT and YW participated pretreatment experiments. ZX completed the microscopic observation. BY, JJ and JQ completed *Miscanthus* sample collection and characterization. LP designed the project, supervised the experiments, interpreted the data and finalized the manuscript. All authors read and approved the final manuscript.

## Supplementary Material

Additional file 1 **Table S1.** Hexoses yield (% cellulose) released from enzymatic hydrolysis after pretreatment. Table S2. Total sugar yield (% dry matter) released from both enzymatic hydrolysis and pretreatment. Table S3. Monosaccharide composition of hemicelluloses (% of total). Table S4. Monosaccharide composition of hemicelluloses (% of total). Table S5. Monosaccharide composition of hemicelluloses (% of total). Table S6. Monomer composition of lignin (% of total). Table S7. Linked phenols of Mfl27 and Mlu12 (μmol/g Dry Matter). Table S8. Correlation coefficient between cellulose CrI and total sugar yield released from both enzymatic hydrolysis and pretreatment. Table S9. Correlation coefficient between cellulose CrI and hexoses yield released from enzymatic hydrolysis after pretreatment. Click here for file
