# Supplementary material for: Hemicelluloses negatively affect lignocellulose crystallinity for high biomass digestibility under NaOH and H2SO4 pretreatments in Miscanthus
Source: Biotechnol Biofuels. 2012 Aug 11;5:58. doi: 10.1186/1754-6834-5-58 (PMC3462114; doi:10.1186/1754-6834-5-58)
Supplement: Additional file 1 — Table S1. Hexoses yield (% cellulose) released from enzymatic hydrolysis after pretreatment. Table S2. Total sugar yield (% dry matter) released from both enzymatic hydrolysis and pretreatment. Table S3. Monosaccharide composition of hemicelluloses (% of total). Table S4. Monosaccharide composition of hemicelluloses (% of total). Table S5. Monosaccharide composition of hemicelluloses (% of total). Table S6. Monomer composition of lignin (% of total). Table S7. Linked phenols of Mfl27 and Mlu12 (μmol/g Dry Matter). Table S8. Correlation coefficient between cellulose CrI and total sugar yield released from both enzymatic hydrolysis and pretreatment. Table S9. Correlation coefficient between cellulose CrI and hexoses yield released from enzymatic hydrolysis after pretreatment. [file 1754-6834-5-58-S1.pdf]

Hemicelluloses negatively affect lignocellulose crystallinity for high biomass digestibility under NaOH and H_2SO_4 pretreatments in *Miscanthus*

Supplementary

Table S1. Hexoses yield (% cellulose) released from enzymatic hydrolysis after pretreatment

Pair	Sample	NaOH						H_2SO_4					
		**0.5%**		**1%**		**4%**		**0.25%**		**1%**		**4%**	
**I**	Mlu26 (H)	54.9±0.9	1.6 *	72.8±0.4	1.6	98.8±1.7	1.3	29.2±0.2	1.5	40.8±1	1.5	47.0±1.0	1.4
	Msi34 (L)	34.7±0.6		44.4±0.6		75.6±0.9		18.9±0.7		26.5±0.7		34.2±0.9	
**II**	Mfl03 (H)	37.1±1.3	1.4	48.6±0.8	1.4	80.6±0.6	1.3	22.9±0.6	2	37.9±0.4	2.1	41.9±0.6	1.7
	Mlu01(L)	26.3±1.5		34.8±0.2		60.5±1.6		11.2±0.7		18.2±0.8		22.5±0.8	
**III**	Mfl40 (H)	33±0.5	1.9	54.4±1.4	1.5	74.6±7.7	1.3	16.2±1.8	1.4	32.4±0.4	2.6	40.8±0.4	1.8
	Msa02 (L)	17.4±1.7		36.5±1.5		58.5±5.7		11.9±0.4		12.7±0.4		22.6±0.2	
**IV**	Msi56 (H)	48.8±3.1	1.4	57.9±0.7	1.1	84.1±6	1.1	27.6±1.1	1.5	43.1±0.9	1.5	51.9±0.3	1.4
	Mfl04 (L)	33.8±2.4		50.8±0.8		77.1±1.1		18.2±0.9		29.1±1.5		38.4±0.6	
**V**	Msa20 (H)	47.3±2.9	2.0	64±1.2	1.8	90.9±1.4	1.2	27.2±1.2	2.7	35.1±1.4	1.2	51.0±0.5	1.6
	Mfl08 (L)	24.2±2.1		35.3±0.4		76.3±1.5		10.3±0.5		28.4±0.9		31.9±1.9	
**VI**	Mfl27 (H)	46.3±1.5	2.1	65.6±3.4	2.2	96.6±3.4	1.8	20.8±1.0	1.8	42.1±0.9	2.0	49.2±1.2	2.1
	Mlu12 (L)	21.8±0.8		30.1±1.5		54.0±1.3		11.3±0.4		21.2±0.8		23.7±0.6	

* Ratio of two sample values at pair

Table S2. Total sugar yield (% dry matter) released from both enzymatic hydrolysis and pretreatment

Pair	Sample	NaOH						H_2SO_4					
		0.5%		1%		4%		0.25%		1%		4%	
I	Mlu26 (H)	30.9±0.2	1.8*	43.7±0.4	1.7	59.0±0.9	1.3	29.3±0.4	1.3	40.2±0.4	1.4	44.1±0.7	1.1
	Msi34 (L)	17.1±0.5		26.5±0.6		46.6±0.6		23.3±0.1		29.7±0.4		38.7±0.6	
II	Mfl03 (H)	19.3±1.4	1.2	28.6±0.9	1.2	49.9±0.1	1.2	26.9±0.2	1.4	37.5±1.2	1.4	40.1±0.4	1.2
	Mlu01 (L)	16.6±0.1		23.9±0.2		42.3±0.8		18.6±0.3		27.0±0.3		34.8±0	
III	Mfl40 (H)	21.8±0.3	2	38.1±0.2	1.6	50.6±2.4	1.3	26.9±0.8	1.5	36.6±0.4	1.5	39.3±0.8	1.2
	Msa02 (L)	11.1±1		24±0.7		39.2±2.2		17.4±0.1		24.7±0.3		33.9±1.3	
IV	Msi56 (H)	36.0±2.5	2.3	47.5±0.5	1.8	63.7±2.5	1.4	33.5±0.8	1.5	46.1±0.7	1.4	52.7±0.7	1.3
	Mfl04 (L)	15.5±0.6		26.8±0.2		45.1±0.9		22±0.2		32.5±0.9		39.2±0.4	
V	Msa20 (H)	24.4±1.1	1.4	37.7±0.9	1.3	52.8±0.5	1.1	28.2±0.5	1.2	37.3±0.4	1.3	44.2±1.6	1.2
	Mfl08 (L)	17.5±0.5		28.5±0.5		49.9±0.3		23±0.8		28.1±0.6		38.4±1.3	
VI	Mfl27 (H)	23.9±1.0	1.5	36.5±1.1	1.4	54.4±1.0	1.2	31.6±0.4	1.5	42.3±1.3	1.5	45.2±0.6	1.1
	Mlu12 (L)	16.5±0.5		26.3±1.1		44.0±0.7		21.0±0.1		29.2±0.6		39.6±0.4	

* Ratio of two sample values at pair

Table S3. Monosaccharide composition of hemicelluloses (% of total)

Pair	Sample	Rha	Fuc	Ara	Xyl	Man	Glu	Gal
IV	Msi56 (H)	0.29% *	ND	13.08%	83.45%	0.20%	1.10%	1.86%
	Mfl04 (L)	0.28%	ND	12.06%	82.34%	0.13%	3.83%	1.36%

* Percentage of total monosaccharides; ND, non-detectable.

Table S4. Monosaccharide composition of hemicelluloses (% of total)

Pair	Sample	Rha	Fuc	Ara	Xyl	Man	Glu	Gal
**V**	Msa20 (H)	0.24% *	ND	12.06%	83.68%	0.59%	1.69%	1.74%
	Mfl08 (L)	0.19%	ND	10.88%	85.17%	0.14%	2.66%	0.95%

* Percentage of total monosaccharides; ND, non-detectable.

Table S5. Monosaccharide composition of hemicelluloses (% of total)

Pair	Sample	Rha	Fuc	Ara	Xyl	Man	Glu	Gal
VI	Mfl27 (H)	0.26% *	ND	12.68%	80.53%	0.14%	4.95%	1.43%
	Mlu12 (L)	0.17%	0.01%	9.88%	87.45%	0.20%	0.99%	1.31%

* Percentage of total monosaccharides; ND, non-detectable.

Table S6. Monomer composition of lignin (% of total)

Pair	Sample	H	G	S	S/G	H/G	S/H
**VI**	Mfl27 (H)	27.79%*	49.02%	23.19%	0.47	0.57	0.83
	Mlu12 (L)	21.41%	45.11%	33.48%	0.74	0.47	1.56

* Percentage of total monomers

Table S7. Linked Phenols of Mfl27 and Mlu12 (μmol/g dry matter)

Linkage	Sample	H-	G-	S-	AV-	AS-	PCA-	FA-	Total
Ester-	Mfl27 (H)	0.13<br>(2.59%)*	0.26<br>(5.12%)	0.09<br>(1.76%)	ND	ND<br>(0%)	1<br>(19.31%)	3.69<br>(71.22%)	5.17
	Mlu12 (L)	0.5<br>(6.77%)	0.99<br>(13.34%)	0.42<br>(5.63%)	ND	0.03<br>(0.36%)	1.48<br>(20.1%)	3.97<br>(53.81%)	7.38
Ether-	Mfl27 (H)	2.86<br>(3.75%)	11.29<br>(14.78%)	9.21<br>(12.05%)	6<br>(0.78%)	13.77<br>(18.02%)	7.56<br>(9.89%)	25.73<br>(33.67%)	76.41
	Mlu12 (L)	3.35<br>(3.48%)	19.46<br>(20.16%)	29.75<br>(30.83%)	6.0<br>(0.63%)	12.59<br>(13.05%)	5.91<br>(6.13%)	19.39<br>(20.1%)	96.49

H-: *p*-Hydroxybenzaldehyde, G-: Vanillin, S-: Syringaldehyde, AV-: Acetovanillone, AS-: Acetosyringone, PCA-: *p*-Coumaric acid, FA-: Ferulic acid, SA-: Sinapic acid;
* percentage of total linked-phenols.

Table S8. Correlation coefficient between cellulose CrI and total sugar yield released from both enzymatic hydrolysis and pretreatment

	NaOH			H_2SO_4		
	**0.50%**	**1%**	**4%**	**0.25%**	**1%**	**4%**
**Raw material CrI**	-.750**	-.748**	-.838**	-.875**	-.839**	-.812**

** significant differences at $p < 0.01$.

Table S9. Correlation coefficient between cellulose CrI and hexoses yield released from enzymatic hydrolysis after pretreatment.

	NaOH			H_2SO_4		
	**0.50%**	**1%**	**4%**	**0.25%**	**1%**	**4%**
**Raw material CrI**	-.735**	-.685*	-.763**	-.580*	-.891**	-.777**

* and ** significant differences at $p < 0.05$ & 0.01, respectively.
